# Whole-genome sequencing data of *Salmonella enterica* subsp. *enterica* serovar Enteritidis strain SSTRA25 isolated from a pediatric bacteremia case in Mosul, Iraq

**DOI:** 10.1016/j.dib.2025.112077

**Published:** 2025-09-17

**Authors:** Shymaa F. Yonis, Sura I. Khudher, Talal S. Salih, Rayan M. Faisal, Ayman M. Khaleel

**Affiliations:** a College of Medicine, University of Mosul, Iraq; b Department of Biology, College of Education for girls, University of Mosul, Iraq; c Department of Medical Physics, College of Science, University of Mosul, Iraq; d Department of Biology, College of Science, University of Mosul, Iraq; e Nineveh Health Department, Mosul, Iraq

**Keywords:** Salmonella sorovar enteritidis, Genome sequence, Antimicrobial resistance genes, Chromosomal point mutations, Pathogenicity

## Abstract

*Salmonella enterica* subsp. *enterica* serovar Enteritidis is a well-known non-typhoidal serovar, commonly associated with foodborne illnesses. Here, we report the draft genome sequence of *Salmonella enterica* subsp. *enterica* serovar Enteritidis strain SSTRA25, isolated from a pediatric patient with bacteremia in Mosul, Iraq. The genome was sequenced using the Illumina NovaSeq 6000 platform. The assembled and annotated genome comprised 4733,231 bp with 40 contigs, and a GC content of 52.12%. It contains 4580 coding sequences (CDSs), 69 tRNAs, 9 rRNAs, 14 ncRNAs, 2 CRISPR arrays, and 368 annotated subsystems. The analysis of antimicrobial resistance genes revealed multiple genes associated with various drug classes, including phenicols, penicillin beta-lactams, cephalosporins, carbapenems, and monobactams, with perfect sequence matches. In addition, chromosomal point mutations linked to antimicrobial resistance were identified with significant sequence similarity. *Salmonella enterica* subsp. *enterica* serovar Enteritidis SSTRA25 showed a high predicted human pathogenicity score (0.941) and carried multiple virulence factors, including SspH2, SopA, SadA, ShdA, MisL, and several flagellar and outer membrane proteins. In the pathogenic landscape, the closest strain was *Salmonella enterica* subsp. *enterica* serovar Holcomb NY_FSL C7–1028, with a Minkowski distance of 0.024804. The genome sequence of *Salmonella enterica* subsp. *enterica* serovar Enteritidis SSTRA25 has been deposited in NCBI under the accession number JBNHMR000000000.

Specifications Table

**Table:** 

Subject	Biology
Specific subject area	Bacteriology, Molecular Microbiology, Genomics
Type of data	Whole Genome Sequence Data in FASTA format, Analyzed, Tables, figures, deposited*.*
Data collection	Genomic DNA of *Salmonella enterica* subsp. *enterica* serovar Enteritidis SSTRA25 was extracted from a 24-hour culture grown on MacConkey agar using the Bacterial DNA Isolation Kit. The DNA library was prepared using the TruSeq Nano DNA Library kit and sequenced on a NovaSeq 6000 Sequencing System from Illumina, USA. Genome was *de novo* assembled to contigs via SPAdes v3.5. General genome statistics were generated using QUAST v5.3.0. Contigs were annotated using Rapid Annotation using Subsystem Technology (RAST) server and the NCBI Prokaryotic Genome Annotation Pipeline (PGAP) v6.9. The acquired antimicrobial resistance genes were identified using CARD database v4.0.1. Chromosomal mutations associated with antimicrobial resistance were identified using ResFinder v4.7.2. The pathogenicity to humans was predicted using PathogenFinder v*2.*
Data source location	*Salmonella enterica* subsp. *enterica* serovar Enteritidis SSTRA25 was isolated from a pediatric bacteremia case in Mosul, Iraq, located at 36.3456° N latitude and 43.1575° E longitude.
Data accessibility	*The draft genome sequence of Salmonella enterica* subsp. *enterica serovar Enteritidis SSTRA25 is available in the NCBI GenBank database under accession number: JBNHMR000000000, with BioProject number: PRJNA1248932 and BioSample number: SAMN47872629.* *Direct URL to data:* https://www.ncbi.nlm.nih.gov/nuccore/JBNHMR000000000 https://www.ncbi.nlm.nih.gov/bioproject/PRJNA1248932 https://www.ncbi.nlm.nih.gov/biosample/SAMN47872629
Related research article	none

## 1 Value of the Data

• The whole-genome sequence of *Salmonella enterica* subsp. *enterica* serovar Enteritidis SSTRA25, isolated from a human in Mosul, Iraq, provides insights into the genetic structure, pathogenicity, and antimicrobial resistance of this clinically important serovar.
• This genomic dataset serves as a valuable reference for comparative genomic studies with other *Salmonella* serovars and related taxa, research on antimicrobial resistance, virulence factors, and evolutionary relationships within the genus.
• These data are valuable to researchers, microbiologists, and public health specialists, enabling comparison of clinical isolates from Iraq with those circulating in neighboring countries, thereby supporting surveillance, diagnostics, and infection control strategies.

## 2 Background

Non-typhoidal *Salmonella* species are known to cause foodborne gastroenteritis as well as severe disseminated infections, which depend on the virulence of the pathogen and the immune status of the host [1]. Research indicates that *Salmonella* can enter the lymphatic system following ingestion, survive and replicate within macrophages, subsequently disseminating to reticuloendothelial organs such as the spleen and bone marrow [2]. Bacteremia caused by non-typhoidal species is rare; however, it is more frequently observed in individuals with predisposing factors such as T-cell deficiencies, hemolytic disorders (including sickle cell disease and malaria), or trauma [3]. Herein, we report a case of *Salmonella enterica* subsp. *enterica* serovar Enteritidis isolated from the blood of a 6-year-old patient with beta-thalassemia who presented with fever and rash at Al-Hadbaa Specialized Hospital in Mosul, Iraq. To date, no cases of bacteremia caused by *Salmonella enterica* subsp. *enterica* serovar Enteritidis have been reported in Iraq and no whole-genome analyses of this pathogen have been conducted. Therefore, this study was undertaken to analyze its genome for features potentially relevant to bacteremia caused by this pathogen*.*

## 3 Data Description

**Fig. 1:**
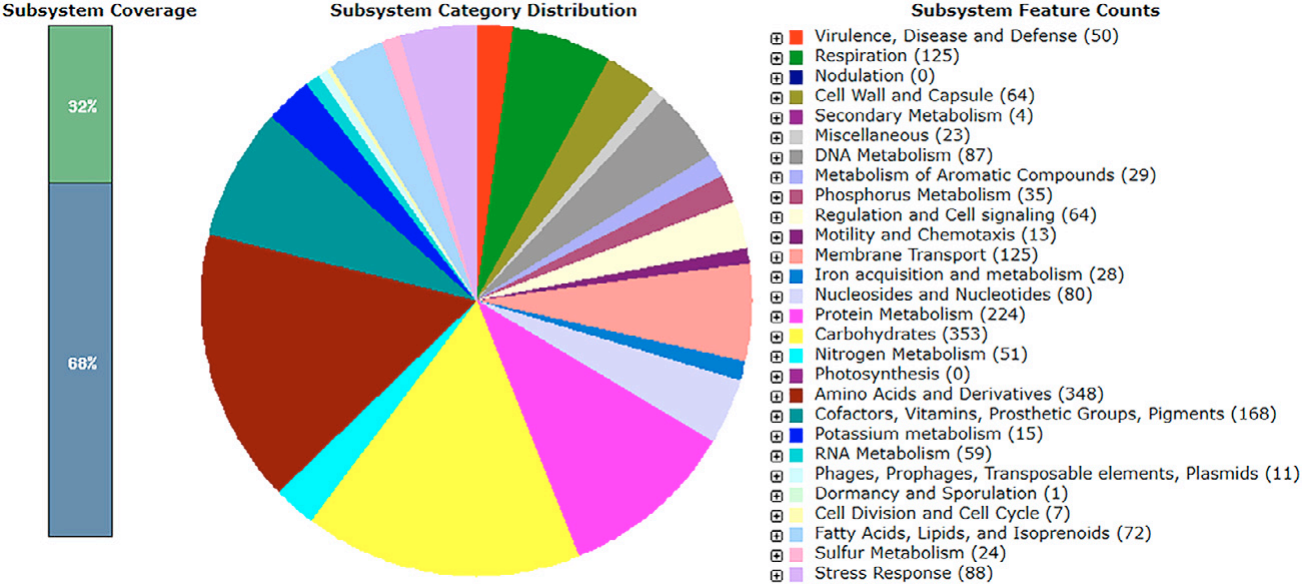
Distribution of subsystem categories in the genome of *Salmonella enterica* subsp. *enterica* serovar Enteritidis SSTRA25.

The genome sequence of *Salmonella enterica* subsp. *enterica* serovar Enteritidis SSTRA25 has a total genome length of 4733,231 base pairs (bp), with a mean DNA GC content of 52.12 %, distributed across 40 contigs larger than 545 bp. General genome features of SSTRA25 are presented in Table 1. The assembled genome exhibited a completeness of 99.68 % and a contamination level of 0.41 %. The annotated genome contains 4580 coding sequences (CDSs), 69 tRNA, 9 rRNA (2 5S, 3 16S, 4 23S), 14 ncRNAs genes, 2 CRISPR arrays and 368 subsystems. A large proportion of the predicted genes within the subsystem categories are associated with the synthesis of carbohydrate metabolism (353), amino acids and their derivatives (265) and protein metabolism (224), followed by cofactors, vitamins, prosthetic groups, pigments (168), then membrane transport (125) and respiration (125). Further genes within the subsystem categories are illustrated in Fig. 1.

**Table 1:** General genome features and assembly statistics of *Salmonella enterica* subsp. *enterica* serovar Enteritidis SSTRA25.

Features	Value
Total length size (bp)	4,733,231
DNA GC content (%)	52.12
Genome coverage (X)	100
Number of contigs	40
Number of contigs (≥ 1000 bp)	38
Longest contig size (bp)	572,046
Shortest contig size (bp)	545
N50	245,215
N90	89,032
L50	6
L90	19

Pairwise genome analysis of *Salmonella enterica* subsp. *enterica* serovar Enteritidis SSTRA25 revealed average nucleotide identity (ANI) and Z-score values of 98.35 % and 0.99999 respectively, when compared with *Salmonella enterica* subsp. *enterica* serovar Enteritidis CVM N48681, its closest strain. In addition, digital DNA-DNA hybridization (dDDH) analysis showed values of 98.28 % (species level) and 80.16 % (subspecies level) between strain SSTRA25 and CVM N48681, both exceeding the recommended thresholds (>70 % for species and >79 % for subspecies delineation).

The genome of *Salmonella enterica* subsp. *enterica* serovar Enteritidis SSTRA25 revealed the presence of antimicrobial resistance genes linked to several drug classes. Genes with perfect matches (100 % sequence identity) were identified for phenicol, penicillin beta-lactam, cephalosporin, carbapenem, and monobactam. Additional resistance genes were detected with strict but lower identity, ranging from 99.58 % (fluoroquinolone) to 38.87 % (glycopeptide) (Table 2). Chromosomal point mutations associated with antimicrobial resistance genes were also identified, including acrB (99.52 %), pmrA (99.7 %), pmrB (99.44 %), gyrA (100 %), gyrB (100 %), parC (99.96 %) and parE (100 %) (Table 3).

**Table 2:** Antimicrobial drug classes, resistance mechanisms, percentage identity, and number of hits identified in *Salmonella enterica* subsp. *enterica* serovar Enteritidis SSTRA25, based on perfect (100 % identity) and strict (99.58–38.87 % identity) matches.

Drug class	Resistance mechanism	Percentage Identity (%)	Number of Hits
Phenicol	Antibiotic efflux	100	1
Penicillin beta-lactam	Antibiotic efflux	100	1
Cephalosporin	Antibiotic efflux	100	1
Carbapenem	Antibiotic efflux	100	1
Monobactam	Antibiotic efflux	100	1
Fluoroquinolone	Antibiotic efflux	99.58	14
Macrolide	Antibiotic efflux	99.05	4
Aminoglycoside	Antibiotic inactivation	97.24	5
Nitroimidazole	Antibiotic efflux	96.39	1
Phosphonic acid	Antibiotic target alteration	95.68	1
Disinfecting agents and antiseptics	Antibiotic efflux	94.66	11
Aminocoumarin	Antibiotic efflux	94.14	1
Tetracycline	Antibiotic efflux	87.71	12
Nucleoside	Antibiotic efflux	86.94	1
Diaminopyrimidine	Antibiotic efflux	85.25	1
Rifamycin	Antibiotic efflux	76.67	9
Peptide	Antibiotic target alteration	62.61	5
Glycopeptide	Antibiotic target alteration	38.87	1

**Table 3:** Chromosomal point mutations identified in the genome sequence of *Salmonella enterica* subsp. *enterica* serovar Enteritidis SSTRA25.

Gene	Identity	Alignment Length/Gene Length	Position in reference	Contig No.	Position in contig	Accession No.
acrB	99.52 %	3150 / 3150	1..3150	3	209,084..212233	CP000026.1
pmrA	99.70 %	669 / 669	1..669	9	60,579..61247	CP055130.1
pmrB	99.44 %	1071 / 1071	1..1071	9	61,257..62327	CP051284.1
gyrA	100 %	2637 / 2637	1..2637	10	20,172..22808	MH933946.1
gyrB	100 %	2415 / 2415	1..2415	20	54,281..56695	CP050716.1
parC	99.96 %	2259 / 2259	1..2259	2	369,665..371923	CP050716.1
parE	100 %	1893 / 1893	1..1893	2	376,680..378572	CP050716.1

The pathogenicity prediction tool assigned a probability score of 0.941. Analysis revealed multiple virulence factors including, E3 ubiquitin-protein ligase SspH2 (SSPH2_SALTY), E3 ubiquitin-protein ligase SopA (SOPA_SALTY), outer membrane protein (A0A5U3Z4M9_SALER), outer membrane protein (A0A6D1IWY1_SALET), autotransporter adhesin SadA (SADA_SALTY), autotransporter adhesin ShdA (A0A608JVW9_SALER), flagellar hook-associated proteins (FLGL_SALTY and FLGK_SALTY), fimbrial usher protein (Q8ZJU1_SALTY), intestinal colonization autotransporter adhesin MisL (A0A5I9EM74_SALGL) and phage tail sheath protein (A0A1G4XFP2_9ENTR). Table 4 lists bacterial species identified as the closest matches in the pathogenicity prediction analysis.

**Table 4:** Top ten closest bacterial species to *Salmonella enterica* subsp. *enterica* serovar Enteritidis SSTRA25 based on pathogenicity prediction analysis.

Bacterial species	Strain	Minkowski Distance	Accession No.
*Salmonella enterica* subsp. *enterica* serovar Holcomb	NY_FSL C7–1028	0.024804	GCF_002106355.1
*Salmonella enterica*	FDAARGOS_709	0.042020	GCF_012273475.1
*Salmonella enterica* subsp. *enterica* serovar Schwarzengrund	MDH-12	0.064420	GCF_003600125.1
*Salmonella enterica* subsp. *enterica* serovar Muenchen	BCW_2736	0.088268	GCF_002064355.1
*Salmonella enterica* subsp. *enterica* serovar Stanley	G117	0.090299	GCF_003945925.1
*Salmonella enterica* subsp. *diarizonae*	19CR6061	0.120665	GCF_020444565.1
*Salmonella enterica* subsp. *enterica* serovar Potsdam	BCW_2762	0.154303	GCF_002065195.1
*Salmonella enterica* subsp. *enterica* serovar Singapore	BCW_2780	0.160421	GCF_002063875.1
*Salmonella enterica*	SM-20	0.160863	GCF_022604135.1
*Escherichia coli*	IBD_EPEC19	0.167274	GCF_022765335.1

## 4 Experimental Design, Materials and Methods

Genomic DNA was extracted from pure colonies of *Salmonella enterica* subsp. *enterica* serovar Enteritidis SSTRA25, grown on MacConkey agar at 37 °C for 24 h, using the Bacterial DNA Isolation Kit (Foregene Co., Ltd, China) according to the manufacturer’s instructions. The Purity and concentration of the extracted DNA were assessed using the NanoDrop spectrophotometer 2000 (Thermo Scientific, USA).

The genomic DNA library was prepared using TruSeq Nano DNA Library kit (Illumina, USA). Whole-genome sequencing of *Salmonella enterica* subsp. *enterica* serovar Enteritidis SSTRA25 was carried out at Macrogen Inc. (Seoul, South Korea) using the Illumina NovaSeq 6000 platform with 2 × 150 bp paired-end reads, yielding approximately 2 × 5.5 gigabases (Gb) of raw data. The adaptors and low quality sequence reads were trimmed using Trimmomatic v0.30 tool [4], with a sliding window trimming quality cutoff of Q20. The trimmed reads were *de novo* assembled into contigs using SPAdes v3.5 [5], with *k-mer* sizes of 21,33,55 and 77, and enabling the ‘–isolate’ option, as recommended for high-coverage datasets.

Genome feature statistics were generated with QUAST v5.3.0 software [6] using default parameters. The completeness and contamination of the assembled genome were assessed with CheckM v1.2.3 [7]. Genome annotation was carried out using both the Prokaryotic Genome Annotation Pipeline (PGAP) v6.9 [8] and the Rapid Annotations using Subsystems Technology (RAST) server [9]. For RAST, the taxonomy identifier 149,539 (*Salmonella* enterica subsp. enterica serovar Enteritidis) and genetic code 11 (bacteria) were applied as annotation parameters.

The average nucleotide identity, BLAST- based, (ANIb) and Z-score values between strain SSTRA25 and CVM N48681 were calculated using JSpecies v5.0.2 software [10]. Digital DNA-DNA hybridization (dDDH) analysis was performed with the Genome-to-Genome Distance Calculator (GGDC) v3.0 web server [11], which estimates genome-to-genome relatedness values for species and subspecies delineation.

Antimicrobial resistance genes were identified using the Comprehensive Antibiotic Resistance Database (CARD) v4.0.1 [12] with default settings. Chromosomal mutations linked to antimicrobial resistance were detected with ResFinder v4.7.2 [13], applying default settings with a 90 % identity threshold, a minimum hit length of 60 %, and Salmonella spp. selected as the reference species. The pathogenic potential of *Salmonella enterica* subsp. *enterica* serovar Enteritidis SSTRA25 in humans was assessed using PathogenFinder v2 [14], based on its whole-genome sequence and applying default parameters.

## Limitations

Not applicable*.*

## Ethics Statement

Ethical approval for this study was granted by the Institutional Ethics Committee of the College of Science, University of Mosul. The protocol code for this study is 7S-112, dated January 20, 2025. Written informed consent was obtained from the patient’s parent in accordance with the Declaration of Helsinki*.*

## CRediT Author Statement

**Shymaa F. Yonis:** Conceptualization, methodology, investigation, data curation, writing. **Sura I. Khudher:** Conceptualization, methodology, investigation, data curation, writing. **Talal S. Salih**: Conceptualization, methodology, investigation, data curation, formal analysis, writing. **Rayan M. Faisal:** Conceptualization, methodology, data curation, writing-review and editing, supervision. **Ayman M. Khaleel:** Conceptualization, methodology, investigation, data curation, writing*.*

## Data Availability

NCBI GenBank databaseSalmonella enterica subsp. enterica serovar Enteritidis strain SSTRA25, whole genome shotgun sequencing project (Original data).
